# The effect of taste alteration on malnutrition and quality of life in patients undergoing chemotherapy

**DOI:** 10.1007/s00520-025-09431-8

**Published:** 2025-04-10

**Authors:** Zeynep Pehlivan Köksal, Nesrin Nural

**Affiliations:** 1 Nursing Department, Faculty of Health Sciences, Recep Tayyip Erdoğan University, Rize, Turkey; 2 Nursing Department, Faculty of Health Sciences, Karadeniz Technical University, Trabzon, Turkey

**Keywords:** Chemotherapy, Malnutrition, Taste alteration, Quality of life

## Abstract

**Objective:**

This descriptive and cross-sectional study was conducted to determine the effect of taste alteration on malnutrition and quality of life in patients undergoing outpatient chemotherapy.

**Materials and methods:**

The sample consisted of 330 patients who underwent chemotherapy in the outpatient chemotherapy unit of a university hospital between March and May 2023. Data were collected through face-to-face interview technique using the “Descriptive Characteristics Form”, “Chemotherapy-Induced Taste Alteration Scale (CI-TAS)”, “Malnutrition Universal Screening Tool (MUST)” and “World Health Organisation Quality of Life Questionnaire (WHOQOL-Tr 8)”. Independent samples *t*-test to compare two groups in normally distributed measurements and analysis of variance (ANOVA) to compare more than two groups (as advanced analysis; least significant difference (LSD) (in cases where the variances are homogeneous), Dunnet C when it was not homogeneous) were used. Pearson correlation analysis and regression analysis were used in correlational inferences.

**Findings:**

It was found that 67.6% of the patients experienced a change in taste. The CI-TAS and WHOQOL-Tr 8 total mean scores of the patients were 2.45 ± 1.17 and 26.01 ± 5.75, respectively, and 21.8% of them were included in the moderate risk group for malnutrition. A significant correlation was found between CI-TAS and MUST and WHOQOL-Tr 8, and CI-TAS had a predictive effect on MUST and WHOQOL-Tr 8 (*R*^2^_adjusted_ = 0.014, *R*^2^_adjusted_ = 0.105).

**Conclusion:**

As the severity of taste alteration intensified in patients, the risk of malnutrition rose, and their quality of life diminished. Consequently, taste alteration is an essential symptom that affects malnutrition and quality of life.

## Introduction

Cancer is one of the major public health problems worldwide [1]. Although the treatment methods employed today have increased the survival rates due to cancer, cancer still ranks second among the diseases that lead to death in the world and in Turkey, and it is predicted that it will rank first in the coming years [2, 3]. According to World Health Organisation data, cancer caused the deaths of 10 million people in 2020 [4].

In cancer treatment, many methods, such as chemotherapy, radiotherapy, surgical treatment, immunotherapy, and targeted therapy, can be utilised either alone or in combination [1]. Chemotherapy, a commonly used method in cancer treatment, is a method where tumours are treated with antineoplastic drugs [5]. These drugs prevent the growth and proliferation of cancer cells as well as destroy healthy cells that proliferate rapidly and can cause physical and psychological symptoms such as pain, fatigue, diarrhoea, altered taste, loss of appetite, and anxiety in patients [6, 7].

Taste alteration is a common symptom in patients who undergo chemotherapy. Studies have reported that taste alteration varies between 45 and 85% in patients who are undergoing chemotherapy [8, 9]. Taste receptor cells have an approximate lifespan of 10 days and a high rate of regeneration. These cells, which have a short lifespan and can proliferate rapidly, are damaged due to the cytotoxic effect of chemotherapy. These damaged cells may cause a reduction in the number of taste receptor cells, impairment of taste perception, alteration of the receptor surface, and, thus, taste alteration [10–12]. Besides chemotherapy, many other factors, such as antibiotic treatment, low fluid consumption, dry mouth due to reduced saliva secretion, oral mucositis, and radiotherapy, can also cause patients to suffer from taste alterations [9].

In the literature, it is reported that taste alteration may cause malnutrition by inducing problems in patients, such as loss of appetite, inadequate energy intake, and weight loss, and may negatively affect their quality of life [13]. Other studies have also found that the diet and food intake of patients who suffer from taste alteration during chemotherapy are disrupted, and the associated risk of malnutrition is high [10, 14]. These clinical problems may cause patients’ treatment plans, leading to prolonged hospitalisation, impaired quality of life, increased treatment costs, and elevated mortality [15]. Due to all these problems, it is thought that the symptoms of taste alteration in patients who undergo chemotherapy should be examined multidimensionally, and its effect on malnutrition and quality of life should be investigated.

Despite the fact that taste alteration is a common symptom in chemotherapy patients, patients frequently do not report it as a problem, and healthcare professionals may overlook it because it is a non-life-threatening symptom. When the studies were examined, they generally examined the side effects of chemotherapy cumulatively or focused on symptoms such as nausea, vomiting, pain, and fatigue. There are a limited number of studies that have examined the effect of taste alteration on malnutrition and quality of life in patients who undergo chemotherapy [13, 15]. This study was conducted to determine the effect of taste alteration on malnutrition and quality of life in patients who undergo chemotherapy.

The research questions:

- What is the prevalence of taste alteration in patients who undergo chemotherapy?
- What is the risk of malnutrition in patients who undergo chemotherapy?
- What is the level of quality of life in patients who undergo chemotherapy?
- Does taste alteration have an effect on malnutrition and quality of life in patients who undergo chemotherapy?

## Material and method

### Study design and setting

The population of this descriptive and cross-sectional research consisted of 2300 patients who were diagnosed with cancer and were treated in the outpatient chemotherapy unit of a university hospital between December 2021 and December 2022. OpenEpi, version 3, publicly available statistical software, was used to calculate the sample size, and the sample size was determined to be at least 330 patients with a significance level of 0.05, a confidence interval of 95%, and the ability to represent the population of 80% [16]. The researchers collected the data by face-to-face interview technique at the convenience of the patients while they were on chemotherapy, and the interview lasted for 10–15 min in each patient.

### Participants

The patients who were on chemotherapy in the outpatient chemotherapy unit of a university hospital between March and June 2023, underwent at least two intravenous chemotherapy protocols, were 18 years of age or older, could communicate verbally, and were voluntary to participate in the study were included in the study. Twenty-nine patients refused to participate in the study. However, new patients continued to be included until the sample size was reached, and the study was completed with 330 patients. The patients who underwent the first cycle of chemotherapy were excluded from the study since the effects of chemotherapy had not yet been noticed.

### Data collection tools

#### Descriptive characteristics form

The Descriptive Characteristics Form consisted of questions about socio-demographic characteristics, cancer diagnosis and treatment properties, taste alterations, and the influencing factors [8–12].

#### Chemotherapy-induced taste alteration scale (CI-TAS)

Kano and Kanda developed the scale in 2013 [17]. Sözeri and Kutlutürkan conducted the validity and reliability study of the scale in Turkey in 2004. While developing the CI-TAS, they aimed to reveal the effects of chemotherapy-induced taste alterations on the individual. The scale consists of three subheadings, four subscales, and 18 items. The scores on the subscales of the scale are utilised when evaluating the scores obtained from the scale. The scores of the subscales are attained by summing the items and dividing by the number of items, and the total score of the scale is attained by summing the scores attained on the subscales and dividing the resultant value by the number of items. The maximum score to be attained from the subscales and the overall scale is five points, while the minimum score is one point. The higher the scores on the scale, the more severe the taste alteration and the more discomfort the individual suffers from. Cronbach’s alpha value of the scale was found to be 0.86 in its validity and reliability study [18].

#### Malnutrition universal screening tool (MUST)

This is a five-step screening method that has been recommended by the European Society of Parenteral and Enteral Nutrition (ESPEN) and the British Association for Parenteral and Enteral Nutrition (BAPEN). The scale consists of three parts (malnutrition, the risk malnutrition, and obesity). The malnutrition risk is classified as low, moderate, or high. The scores on the categories of ‘Body Mass Index (BMI)’, ‘History of Unexpected Body Weight Loss’, and ‘Acute Disease Effects’ are utilised for classification [19].

#### World health organisation quality of life questionnaire (WHOQOL-Tr 8)

The World Health Organisation Quality of Life (WHOQOL) Questionnaires are scales that have been developed through studies conducted simultaneously in many centres. The scale is a quality-of-life instrument consisting of eight items formed by taking certain items from the scales WHOQOL-Bref and WHOQOL- 100. Two of these questions consist of the overall health and general quality of life questions of WHOQOL, and the other six questions consist of physical, spiritual, social, and environmental factors. The answer options are of the five-point Likert type. The lower and upper values of the answer options range from “none” to “totally”. The higher the score, the better the quality of life. The validity and reliability study of the Turkish version of the WHOQOL-Tr 8 for Turkish society and culture (0.85) was conducted by Eser et al. in 2010 [20, 21].

### Data analysis

The data obtained from the study were analysed using the Statistical Package for the Social Sciences (SPSS) for Windows 22 (IBM, 172.16.7.120) software. In the data assessment, numbers, percentage distributions, minimum and maximum values, mean, and standard deviations were used. Independent samples *t*-test to compare two groups in normally distributed measurements and analysis of variance (ANOVA) to compare more than two groups (as advanced analysis; least significant difference (LSD) (in cases where the variances are homogeneous), Dunnet C when it was not homogeneous) were used. Pearson correlation analysis and regression analysis were used in correlational inferences. Cronbach’s alpha coefficient was calculated to determine the internal consistency of the scale items. Kurtosis and skewness coefficient calculations were used to calculate the normality distribution of the data.

### Ethical considerations

Before beginning the study, written permission and ethics committee approval (Date/Decision No. 30/03/2023–29) were obtained from the institution where the research was conducted. Permission of the authors who conducted the reliability and validity of the scales was obtained via e-mail for the use of the scales. Furthermore, the patients included in the study were informed about the purpose of the study and gave their verbal consent based on the principles of voluntariness and willingness. During the research and publication process, the rules of research and publication ethics, principles of the Declaration of Helsinki, and ethical principles were followed.

## Results

The findings of the study indicated that 57.6% of the patients were 60 years of age or older, 51.8% were female, 37% were obese, and 79.1% were married. 50.6% of the patients graduated from primary school, 47.6% lived in a district, 47.6% were smokers, 27.6% had breast cancer, 39.7% were undergoing chemotherapy for five cycles or less, 40% were on chemotherapy once every 2 weeks, and 52.7% were not receiving any additional therapy (Table 1).

**Table 1:** Distribution of patients identifier information

İnformation		Number	Percent
Age (61.06 ± 10.98 year)	40 years and below	13	3.9
	41–59 years old	127	38.5
	60 years and above	190	57.6
Gender	Female	171	51.8
	Male	159	48.2
Body mass index	Underweight	7	2.1
	Normal weight	88	26.7
	Overweight	113	34.2
	Obese	122	37.0
Marital status	Married	261	79.1
	Single or widow	69	20.9
Educational level	Illiterate	37	11.2
	Literate	28	8.5
	Primary school	167	50.6
	Secondary school	25	7.6
	High school	51	15.5
	University and higher	22	6.6
Smoking	Yes	29	8.8
	No	157	47.6
	Quitted	144	43.6
Type of cancer	Breast cancer	107	32.3
	Gastrointestinal tract cancer	85	25.8
	Lung cancer	71	21.5
	Gynaecologic cancer	25	7.6
	Hematologic cancer	22	6.7
	Urinary tract cancer	20	6.1
Number of CT* cycles	≤ 5 cycles	131	39.7
	6–10 cycles	129	39.1
	≥ 11 cycles	70	21.2
Frequency of CT*	Once a week	83	25.2
	Once every 2 weeks	132	40.0
	Once every 3 weeks or more	115	34.8
Status of receiving any treatment in addition to CT*	Yes**	156	47.3
	No	174	52.7

^*^Chemotherapy; **radiotherapy, targeted therapy, immunotherapy

The total mean scores of the patients were 2.45 ± 1.17 on the CI-TAS, 26.01 ± 5.75 on the WHOQOL-Tr 8, and 0.58 ± 0.82 on the MUST. 21.8% of the patients were involved in the moderate risk group in terms of malnutrition. Cronbach’s alpha coefficient was 0.96 and 0.87 on the CI-TAS and WHOQOL-Tr 8 (Table 2).

**Table 2:** Total scale mean scores of the patients

Scales	Number	Min	Max	Ort. ± SS	Cronbach’s *α*
CI-TAS*	330	1.00	5.00	2.45 ± 1.17	0.96
WHOQOL-Tr 8**	330	10.00	40.00	26.01 ± 5.75	0.87
MUST***	330	0	4.00	0.58 ± 0.82	-
According to cut-off point of MUST Scale	**Number**	**Percent**	-	-	-
Low risk	202	61.2	-	-	-
Moderate risk	72	21.8	-	-	-
High risk	56	17.0	-	-	-

^*^Chemotherapy-Induced Taste Alteration Scale; **World Health Organisation Quality of Life Questionnaire; ***Malnutrition Universal Screening Tool

The patients expressed that 67.6% of them experienced taste alterations, 77.9% had mouth sores, 88.5% had dry mouth, 83.9% suffered from gingival sensitivity, and 17.9% had gingival pain. 92.1% of the patients did oral care; 65.4% brushed their teeth; 9.1% rinsed their mouth with vinegar; 11.8% rinsed their mouth with carbonated water; and 49.1% gargled. 45.5% of the patients maintained a good appetite, 65.5% consumed 1500 ml or more of fluid daily, and 54.8% suffered from weight loss after chemotherapy (Table 3).

**Table 3:** Comparison of the scale scores of the patients according to their descriptive and taste alteration-related characteristics

		n(%)	CI-TAS	Test p-value	WHOQOL-TR 8	Test p-value
			Mean ± SD.		Mean ± SD.	
Age	40 years and below	13 (3.9)	2.67 ± 1.26	*F* = 0.899 *p* = 0.408	28.00 ± 5.61	*F* = 0.811 *p* = 0.445
	41–59 years old	127 (38.5)	2.54 ± 1.20		25.91 ± 5.61	
	60 years and above	190 (57.6)	2.38 ± 1.15		25.94 ± 5.86	
Gender	Female	171 (51.8)	2.81 ± 1.15	*t* = 6.037 *p* = **0.000**	24.74 ± 5.53	*t* = −4.255 ***p* = 0.000**
	Male	159 (48.2)	2.07 ± 1.07		27.37 ± 5.69	
Type of cancer	Breast cancer^a^	107 (32.3)	2.91 ± 1.12	*F* = 4.421 *p* = **0.000**	24.67 ± 5.66	*F* = 3.326 ***p* = 0.003**
	Gastrointestinal tract^b^	85 (25.8)	2.26 ± 1.16		25.62 ± 5.95	
	Lung^c^	71 (21.5)	2.23 ± 1.08		27.82 ± 5.21	
	Gynaecological system	25 (7.6)	2.74 ± 1.28		23.92 ± 5.28	
	Hematologic system^e^	22 (6.7)	2.32 ± 1.20		27.23 ± 5.64	
	Urinary system^d^	20 (6.1)	1.92 ± 1.15		27.90 ± 5.35	
Number of CT* cycles	≤5 cycles	131 (39.7)	2.41 ± 1.13	*F* = 0.294 *p* = 0.746	26.96 ± 5.95	*F* = 3.021 *p* = 0.050
	6–10 cycles	129 (39.1)	2.45 ± 1.20		25.40 ± 5.82	
	≥ 11 cycles	70 (21.2)	2.54 ± 1.23		25.34 ± 5.04	
Frequency of CT*	Once a week	83 (25.2)	2.74 ± 1.22	*F* = 2.387 *p* = 0.069	25.18 ± 5.64	*F* = 2.146 *p* = 0.094
	Once every 2 weeks	132 (40.0)	2.31 ± 1.14		25.60 ± 5.94	
	Once every 3 weeks or more	115 (34.8)	2.42 ± 1.16		27.08 ± 5.52	
Additional treatment to CT*	Yes**	156 (47.3)	2.44 ± 1.18	*t* = −0.248 *p* = 0.805	26.85 ± 5.48	*t* = −2.544 ***p* = 0.011**
	No	174 (52.7)	2.47 ± 1.17		25.25 ± 5.64	
Taste alteration	Yes	223 (67.6)	2.95 ± 1.05	*t* = 16.913 *p* = **0.000**	25.09 ± 5.44	*t* = −4.198 ***p* = 0.000**
	No	107 (32.4)	1.42 ± 0.59		27.93 ± 5.92	
Oral mucositis	Yes	73 (22.1)	2.97 ± 1.15	*t* = 4.353 *p* = **0.000**	24.41 ± 5.79	*t* = −2.717 ***p* = 0.007**
	No	257 (77.9)	2.31 ± 1.14		26.46 ± 5.67	
Dry mouth	Yes	38 (11.5)	3.45 ± 1.03	*t* = 5.832 *p* = **0.000**	24.68 ± 6.09	*t* = −1.513 *p* = 0.131
	No	292 (88.5)	2.32 ± 1.13		26.18 ± 5.68	
Gingival sensitivity	Yes	53 (16.1)	3.15 ± 1.15	*t* = 4.853 *p* = **0.000**	24.40 ± 6.22	*t* = −2.242 ***p* = 0.026**
	No	277 (83.9)	2.32 ± 1.13		26.32 ± 5.62	
Gingival pain	Yes	59 (17.9)	3.15 ± 1.13	*t* = 5.257 *p* = **0.000**	24.71 ± 5.95	*t* = −1.920 *p* = 0.056
	No	271 (82.1)	2.30 ± 1.13		26.29 ± 5.68	
Doing oral care	Yes	304 (92.1)	2.45 ± 1.17	*t* = −0.006 *p* = 0.995	26.17 ± 5.78	*t* = 1.791 *p* = 0.074
	No	26 (7.9)	2.46 ± 1.18		24.08 ± 5.17	
Brushing tooth	Yes	213 (64.5)	2.41 ± 1.17	*t* = 0.576 *p* = 0.565	26.36 ± 5.95	*t* = 1.485 *p* = 0.139
	No	117 (35.5)	2.33 ± 1.16		25.38 ± 5.33	
Gargling	Yes	162 (49.1)	2.44 ± 1.20	*t* = −0.159 *p* = 0.874	25.98 ± 5.89	*t* = −0.105 *p* = 0.917
	No	168 (50.9)	2.46 ± 1.15		26.04 ± 5.63	
Rinsing the mouth with vinegar	Yes	30 (9.1)	3.16 ± 1.16	*t* = 3.534 *p* = **0.000**	26.17 ± 4.72	*t* = 0.157 *p* = 0.875
	No	300 (90.9)	2.38 ± 1.15		25.99 ± 5.85	
Rinsing the mouth with carbonated water	Yes	39 (11.8)	3.06 ± 1.09	*t* = 3.511 *p* = **0.001**	26.44 ± 5.63	*t* = 0.493 *p* = 0.622
	No	291 (88.2)	2.37 ± 1.16		25.95 ± 5.77	
Daily fluid consumption (millilitres)***	≤500 ml	41 (12.4)	2.78 ± 1.38	*F* = 2.879 *p* = 0.058	25.32 ± 6.38	*F* = 0.436 *p* = 0.674
	500–1500 ml	73 (22.1)	2.58 ± 1.15		25.85 ± 5.56	
	≥1500 ml	216 (65.5)	2.35 ± 1.13		26.19 ± 5.70	
Appetite level	Poor^e^	49 (14.8)	3.42 ± 1.11	*F* = 35.277 *p* = **0.000**	21.31 ± 5.25	*F* = 31.305 ***p* = 0.000**
	Moderate^f^	73 (22.1)	2.92 ± 0.85		25.07 ± 4.28	
	Good^g^	150 (45.5)	2.24 ± 1.13		26.19 ± 5.15	
	Very good^h^	58 (17.6)	1.60 ± 0.82		30.71 ± 5.69	
Weight loss after CT*	Yes	181 (54.8)	2.58 ± 1.21	*t* = 2.221 *p* = **0.027**	26.22 ± 5.70	*t* = −3.821 ***p* = 0.000**
	No	149 (45.2)	2.30 ± 1.11		25.13 ± 5.92	

*Chemotherapy; **radiotherapy, targeted therapy, immunotherapy; a > b, c, d, e; g, h > e, f; ***calculated by glass and one glass is assumed to be 200 ml; t: t-test in independent groups; F: analysis of variance

There was a statistically significant difference between gender, cancer type, additional treatment, taste alteration, oral mucositis, dry mouth, gingival sensitivity and pain, rinsing the mouth with vinegar and carbonated water, appetite level and weight loss after chemotherapy, and the CI-TAS total mean score (*p* < 0.05). The CI-TAS total mean score was higher in women than in men, higher in patients with breast cancer than in patients with gastrointestinal, lung, urinary, and hematologic cancers, and lower in patients with a good or very good appetite than in patients with a poor or moderate appetite. No statistically significant difference was found between age, the number of chemotherapy cycles, frequency of chemotherapy, additional treatment to chemotherapy, doing oral care, brushing teeth, gargling, and daily fluid consumption, and the CI-TAS total mean score (*p* > 0.05) (Table 3).

The difference in the WHOQOL-TR 8 total mean scores was statistically significant according to gender, type of cancer, additional treatment to chemotherapy, taste alteration, oral mucositis, gingival sensitivity and pain, and appetite level (*p* < 0.05). The total mean score of the WHOQOL-TR 8 was higher in patients with lung cancer than in patients with gynaecologic, gastrointestinal, and breast cancer, and in patients with a good or very good appetite than in patients with a poor or moderate appetite. The difference between the WHOQOL-TR 8 total mean score and dry mouth, oral mucosal pain, doing oral care, brushing teeth, gargling, rinsing the mouth with vinegar and carbonated water, daily fluid consumption, and weight loss after chemotherapy was not statistically significant (*p* > 0.05) (Table 3).

There was a statistically significant, positive, and weak correlation between CI-TAS and MUST scores and a negative and weak correlation between CI-TAS and WHOQOL-Tr 8 scores (*p* < 0.05). As the CI-TAS scores increased, the MUST scores increased and the WHOQOL-Tr 8 scores decreased. There was a statistically significant, negative, and weak correlation between WHOQOL-Tr 8 and MUST scores (*p* < 0.05). As the WHOQOL-Tr 8 scores increased, the MUST scores decreased (Table 4).

**Table 4:** The correlation between total scores of CI-TAS, MUST, and WHOQOL-TR 8

		**CI-TAS**	**MUST**	**WHOQOL-Tr 8**
CI-TAS	*r*	-	0.129	− 0.329
	*p*	**-**	**0.019**	**0.000**
	*n*	-	330	330
WHOQOL-Tr 8	*r*	-	− 0.250	-
	*p*	**-**	**0.000**	**-**
	*n*	-	330	-
Pearson correlation analysis				

A significant correlation was found between the CI-TAS and MUST scores (*R* = 0.129, *R*^2^_adjusted_ = 0.014, *F*_(1.328)_ = 5.563; *p* = 0.019). The CI-TAS total score accounted for 1.4% of the change in the MUST. The regression equation that predicts the MUST scores was as follows: MUST = (0.091 × the CI-TAS score) + (0.353). A significant correlation was found between the CI-TAS and WHOQOL-Tr 8 total scores (*R* = 0.329, *R*^2^_adjusted_ = 0.105, *F*_(1.328)_ = 39.700; *p* = 0.000). The CI-TAS total score accounted for 10.5% of the change in the WHOQOL-Tr 8 total score. The regression equation that predicted the WHOQOL-Tr 8 total score was as follows: the WHOQOL-Tr 8 score = (− 1.611 × the CI-TAS score) + (29.963) (Table 5).

**Table 5:** Multiple regression analysis between MUST and WHOQOL-Tr 8 total score and CI-TAS

**Beta**		**Standard error**	**Standard beta**	***t***	***p***	**Confidence Interval of 95%**	
MUST							
Constant coefficient	0.353	0.104	-	3.385	**0.001**	0.148	0.559
CI-TAS	0.091	0.038	0.129	2.359	**0.019**	0.015	0.166
WHOQOL-Tr 8							
Constant coefficient	29.963	0.695		43.096	**0.000**	28.595	31.330
CI-TAS	− 1.611	0.256	− 0.329	− 6.301	**0.000**	− 2.114	− 1.108

## Discussion

Taste alteration in patients who undergo chemotherapy may lead to impaired nutritional condition and quality of life, disruptions in the treatment plan, prolonged hospitalisation, and elevated treatment costs and mortality [15]. This study determined the effect of taste alteration on malnutrition and quality of life in patients who underwent chemotherapy, and the findings were discussed in this section along with the literature.

The findings of this study showed that taste alteration was observed in two-thirds (67.6%) of the patients who underwent chemotherapy and was of moderate severity (2.45 ± 1.17). Previous studies reported that the prevalence of taste alteration in patients who underwent chemotherapy varied between 20 and 85% [8, 9, 11, 22–24]. Also, similar to the results of this study, different studies reported that the severity of taste alteration was moderate (2.49 ± 1.36; 2.28 ± 0.83) [13, 25]. According to the findings from this study, taste alteration is one of the important symptoms in two-thirds of patients who undergo chemotherapy and are at moderate severity.

Loss of appetite and the associated malnutrition are common symptoms in patients who undergo chemotherapy. This study revealed that one-fifth of the patients (21.8%) were in the moderate-risk group for malnutrition. In a study, it was found that one-fourth (27%) of patients who underwent chemotherapy suffered from moderate malnutrition [26]. In a prospective study conducted on cancer patients who underwent chemotherapy, it was found that one-fourth (26.5%) of patients suffered from mild malnutrition in the first stage and one-fifth (21.9%) in the following years [27]. According to this finding, one-fifth of patients who undergo chemotherapy were in the moderate-risk group for malnutrition.

In the literature, it is reported that cancer patients’ quality of life gets significantly impaired due to the many negative experiences associated with both disease and treatment [28, 29]. In this study, it was found that the quality of life of patients who underwent chemotherapy was at a moderate level (26.01 ± 5.75). The related studies on patients who underwent chemotherapy in Turkey found that their quality of life was at moderate level [30, 31]. Studies conducted in different countries have found that patients who underwent chemotherapy have a poor quality of life [32, 33]. According to these findings, it can be concluded that chemotherapy impaired the patient’s quality of life.

Female patients suffered from a higher severity of taste alteration and a lower quality of life. Similar to these results, studies conducted on patients who were diagnosed with lung cancer found that there was a significant correlation between gender and taste alteration, and female patients suffered from taste alteration more intensely and severely compared to male patients [25, 34]. It is considered that the female gender may be an important factor affecting taste alteration and quality of life in patients who are on chemotherapy.

In the literature, it is reported that taste alteration was affected by many factors, such as cancer type, number of chemotherapy cycles, and frequency of chemotherapy [35]. This study found that patients who were diagnosed with breast and gynaecologic cancer suffered from a higher severity of taste alteration and a lower quality of life. Contrary to this study, a different study reported no significant correlation between cancer type and taste alteration [18]. Different studies also determined that the severity of taste alteration intensified as the number of chemotherapy cycles and frequency of chemotherapy also increased [35, 36]. The effect of cancer type on taste alteration may be attributed to the prevalence of breast and gynaecologic cancers in the female gender and the higher severity of taste alteration and lower quality of life in female patients.

The literature has reported that taste alteration in patients who undergo chemotherapy may develop due to many factors, such as low fluid consumption, dry mouth caused by reduced saliva secretion, and oral mucositis [9]. This study revealed that patients with oral mucositis, dry mouth, gingival sensitivity, and gingival pain suffered significantly higher severity of taste alteration and lower quality of life. Also, those who consumed less than 500 ml of fluid per day suffered from a higher severity of taste alteration, but there was no significant correlation between them. A study reported that reduced saliva secretion in cancer patients, for any reason, diminished taste perception and induced taste alteration [24]. A different study, contrary to this study, reported that the presence of oral problems (dry mouth, oral mucositis) in patients who underwent chemotherapy was not a significant factor affecting the perception of taste alteration [13]. The findings of this study suggest that oral problems (oral mucositis, dry mouth, gingival sensitivity, and gingival pain) in patients who underwent chemotherapy intensified the severity of taste alteration and impaired quality of life, but fluid consumption had no effect.

In this study, it was found that the severity of taste alteration was significantly higher in patients who rinsed their mouths with vinegar and carbonated water. These findings suggested that patients who suffered from taste alterations felt helpless and pursued different approaches (vinegar and carbonated water) in the management of taste alterations, but they failed to find a solution.

This study revealed that half of the patients (54.8%) lost weight after chemotherapy, and patients who lost weight and had poor appetite had significantly higher severity of taste alteration and lower quality of life. Also, as the severity of taste alteration increased, the risk of malnutrition increased, and their quality of life impaired. Taste alteration was a symptom that negatively affected malnutrition and quality of life. As the risk of malnutrition rises, the quality of life impairs. Likewise, previous studies determined that taste alteration in patients who undergo chemotherapy negatively affects the diet and food intake of patients and raises the risk of malnutrition [10, 14, 39]. Another study found that taste alterations in patients who underwent chemotherapy resulted in a loss of appetite and significantly impaired quality of life [37]. Similar to these results, it was also found that the prevalence of malnutrition in patients who underwent chemotherapy was high, and the associated quality of life was poor [38]. According to these findings, it may be asserted that taste alterations in patients who undergo chemotherapy represent an important factor affecting malnutrition and quality of life.

One of the strengths of this study is its multidimensional assessment of how taste alterations affect malnutrition and quality of life by using scales with high Cronbach’s alpha coefficients and its contribution to the literature. Another strength is the completion of the study with a sufficient number of patients and the analysis with parameters selected based on the literature. The limitation of the study is the subjective evaluation of the prevalence of taste alteration and the outcomes based on patient statements, as well as the lack of following up the patients throughout the process.

### Limitations of the research

One of the limitations of this study is the assessment of the effect of taste alteration on malnutrition and quality of life only once and the lack of follow-up in the ongoing process. This is because no comparison has been made in terms of taste changes before and after receiving chemotherapy. Another limitation is that patients may have suffered from more than one symptom simultaneously, and the way they perceived and the meaning they attributed to this symptom may have affected their responses. Finally, since no objective data collection tool was available to demonstrate the prevalence of taste alteration, the subjective data of the patients was used in the study.

## Conclusions

The study, risk of malnutrition raised, and the quality of life impaired as the severity of taste alteration intensified, and consequently, taste alteration was an important symptom affecting malnutrition and quality of life. It is recommended to conduct future studies where the prevalence of taste alteration is evaluated and followed up with objective data collection tools. It is also recommended to conduct randomised controlled trials that can reduce the incidence and severity of this common symptom. In this context, it is important to assess the effect of preventive or therapeutic nursing care and practices on the risk of malnutrition, quality of life, treatment adherence, hospitalisation, and mortality in future studies.

## Data Availability

No datasets were generated or analysed during the current study.
